# New Efficient Adsorbent Materials for the Removal of Cd(II) from Aqueous Solutions

**DOI:** 10.3390/nano10050899

**Published:** 2020-05-08

**Authors:** Aurelia Visa, Bianca Maranescu, Lavinia Lupa, Luminita Crisan, Ana Borota

**Affiliations:** 1 “Coriolan Dragulescu” Institute of Chemistry, 24 M. Viteazul Ave, 300223 Timişoara, Romania; avisa@acad-icht.tm.edu.ro (A.V.); bmaranescu@acad-icht.tm.edu.ro (B.M.); lumi_crisan@acad-icht.tm.edu.ro (L.C.); 2 Faculty of Industrial Chemistry and Enviromental Engineering, University Politehnica Timisoara, 2 Piata Victoriei, 300006 Timişoara, Romania; lavinia.lupa@upt.ro

**Keywords:** coordination networks, adsorption, wastewaters, semiempirical PM3 method

## Abstract

The rapid increase of industrial activities leads to serious environmental pollution, especially, in aqueous systems and particularly with heavy metals. Cadmium, one of the most poisonous elements, is rapidly accumulated in the human body, therefore, the efficient removal of cadmium ions from wastewater is an urgent need. Coordination networks (CNs) and its subdivision metal-organic frameworks (MOFs), are structured porous composites which present various special properties. In this work two CNs were used as adsorbent materials for the removal of Cd(II) ions from aqueous solutions. By the reaction of CoSO_4_·7H_2_O and NiSO_4_·7H_2_O with *N*,*N*-bis(phosphonomethyl)glycine (Gly) in hydrothermal conditions two CNs—Co–Gly and Ni–Gly— were synthesized, respectively. Cadmium adsorption onto the studied CNs was conducted in batch mode, and the effect of pH, initial concentration, contact time, temperature and sorbent weight on the sorption process were investigated. Parametric Method 3 (PM3)semi-empirical analyses of the CNs’ structural properties were performed in order to predict the adsorption properties. For this reason, two octahedral models were calculated and computational predictions were compared with the experimental results. Both computational and experimental adsorption studies found that Ni–Gly presents higher affinity for cadmium ions. Moreover, the adsorbent materials can be readily regenerated and recycled without significant loss of cadmium uptake capacity.

## 1. Introduction

The major drawbacks of the industrial development are the quantity and diversity of wastes which are discharged in the environment. One of the most dangerous groups of inorganic pollutants is represented by the heavy metals since these are not susceptible to biological degradation [1,2].

Porous activated carbons, zeolites, bio-adsorbent materials and carbon nanotubes are extensively used as adsorbents for the removal of heavy metals [3]. However, practical applications of these materials are limited by their low adsorption capacities, low efficiencies, or high cost. With the rapid progress in new material development, metal-organic frameworks (MOFs) and coordination networks (CNs) have received increasing attention in recent years [4]. MOFs are mostly constructed based on metal organic carboxylic derivatives from metal ion nodes linked by organic linkers to form a variety of 1D chain, 2D layer and a three-dimensional (3D) crystal structures with micropores. Phosphonate coordination networks are fast gaining an essential position amongst the families of CNs materials. To expand the adsorption capacity of CNs in a quite large range of pH, it is suggested to choose CNs which are stable in water media [5]. Compared with other adsorbent materials, the main advantages of MOFs and CNs in adsorption processes are their large specific surface area, well ordered unique structures, stable and homogeneous pores of specific sizes. Certainly, MOFs demonstrate good absorbance capacities for a high variety of species that include heavy metals [6,7], drugs [8,9] and dyes [10] from wastewaters.

Cadmium contamination results from many sources such as metal plating, iron and steel production, mining operations, phosphate fertilizer manufacture and use [11]. Even at a low dosage, it can be harmful to both human health and the environment [12]. For this reason, a lot of treatment methods like ion exchange, precipitation, filtration, oxidation-reduction, membrane separation, and adsorption have been developed for the treatment of wastewaters with heavy metal content [13,14,15]. The most economical, feasible and selective method for heavy metal removal from aqueous solutions is the adsorption technique [16,17]. Therefore, researchers are focused on the development of new and more efficient adsorbent materials ranging from natural substances to highly selective synthetic systems to be used as hazardous metal adsorbents [2,18,19,20,21,22,23]. Heavy metal ions, even at small concentrations, are extremely toxic to alive organisms, because they are non-biodegradable, and they tend to accumulate in the environment. 

To the best of our knowledge, so far only a few studies have reported the removal of cadmium ions from aqueous solutions throughadsorption onto MOF materials, in which some composites such as cyclodextrin metal-organic framework-based nanoporous carbon [24] and sulfonated MOF loaded onto iron oxide nanoparticles (Fe_3_O_4_@MOF235(Fe)–OSO_3_H were used [25]. The preparation of these materials involves the use of greater quantities of reagents and many preparation steps, which lead to an increase of the production costs. 

Taking into account the International Union of Pure and Applied Chemistry (IUPAC) nomenclature and terminology recommendation [26] that coordination networks (CNs) are a subdivision of coordination polymers and MOFs a further subset of coordination networks, we will henceforth name our materials as CNs.

In the present paper, the use of two coordination networks based on cobalt and nickel were used as adsorbent materials in the removal process of cadmium ions from aqueous solutions. The structure, morphology, and properties of materials were investigated by Fourier-transform infrared spectroscopy (FTIR), scanning electron microscopy (SEM) and thermal gravimetric analysis (TGA), which were previously described [27,28]. Inspired by these adsorption properties, we performed PM3 semiempirical analyses of structural properties to predict and understand better some special properties of these compounds. Therefore, octahedral models were calculated for networks containing Ni^2+^ and Co^2+^ ions and *N*,*N*-bis(phosphonomethyl)glycine. Bond lengths/angles, torsion angles and partial charges for the central metal ions Ni and Co coordination networks are compared.

## 2. Materials and Methods 

All chemicals were of reagent grade quality achieved from commercial sources and used without further purification. Ni(CH_3_COO)_2_·4H_2_O and Co(NO_3_)_2_·6H_2_O were purchased from Merck (Milipore, Darmstadt, Germany), *N*,*N*-bis(phosphonomethyl)-glycine and Sodium hydroxide (Sigma Aldrich Chemie GmbH (München, Germany) and urea from Alfa Aesar (Karlsruhe, Germany). 

### 2.1. Instrumentation

The specific surface area together with a pore volume of Co–Gly and Ni–Gly were measured with an ASAP 2020 BET surface area analyzer (Micrometrics, Micrometrics Instrument Corporation, Norcross, GA, USA) by cold nitrogen adsorption. SEM images were registered with a FEG 250 microscope (Quanta, Field Electron and Iron Company (FEI), Hillsboro, OR, USA), equipped with an EDAX/ZAF quantifier. Cadmium ion concentrations were measured via a SpectrAA 280 FS atomic adsorption spectrophotometer (Varian, Melbourne, Australia). Thermal analysis (TG-DTA) data were recorded on an SDT-Q600 analyzer from TA Instruments (New Castle, DE, USA). A Diamond thermogravimetric analyzer (Perkin Elmer, New York, NY, USA) was used applying temperatures between 30 and 680 °C under a N_2_ flow increasing the heating at a rate of 10 °C/min. The adsorption studies of investigated materials were made in batch mode using a SW23 shaker bath (Julabo Labortechnik GmbH, Sellbach, Germany).

### 2.2. Materials Synthesis 

A 250 mL Erlenmeyer flask was filled with Ni(CH_3_COO)_2_·4H_2_O (50.0 mmol) or Co(NO_3_)_2_·6H_2_O (50.0 mmol) and bidistilled water (50 mL). The materials were stirred with a constant speed of 1000 rpm until a clear (green or violet) solution was formed. In another flask *N*,*N*-bis(phosphonomethyl)-glycine, urea (50.0 mmol), and bidistilled water (50 mL) were mixed in the same conditions till a incolor clear solution was formed. Both solutions were mixed in a 250 mL Erlenmeyer flask and the pH was adjusted to 4.5 in the case of Ni containing synthesis and 2.8 in the case of Co containing synthesis with an aqueous solution of NaOH (0.1 M). Then the clear green or violet solution was heated in an oil-bath at 80°C for 75 h, unperturbed. After 75 h heating crystalline green (Ni–Gly) and violet crystals (Co–Gly) materials precipitated and were isolated by filtration and finally air dried (yield: 52–75%) [27,28,29].

### 2.3. Adsorption Studies

All the adsorption studies were conducted in batch mode. In the first step the influence of the pH upon the adsorption capacity of Co–Gly and Ni–Gly was determined. For each experiment 25 mL of a solution containing 30 mL of cadmium ions were treated with 0.05 g of adsorbent material for 1 h at a constant speed of 200 rpm, using a Julabo SW23 shaker bath. After 1h of reaction, the samples were filtered and the residual concentration of Cd(II) ions was analyzed in the filtrate by atomic adsorption spectrophotometer. The pH adjustment of the solution was done using 1.0 M NaOH or 1.0 M HCl and was measured using a pH-meter (Mettler Toledo, Giessen-Germany). 

The adsorption capacity of the studied materials in the removal process of Cd(II) was calculated according to the mass balance (Equation (1)):

(1) $$q=\frac{\left(C_{0}-C_{e}\right)\cdot V}{m}$$

where *q* is the amount of Cd(II) adsorbed (mg/g); *C*_0_ and *C_e_* represents the initial and equilibrium concentration of Cd(II) in the solutions (mg/L), respectively. *V* represents the solution volume (L) and *m* represents the adsorbent mass (g) used in the experiments.

To study the effect of contact time on adsorption, further experiments were carried out using the same S:L ratio, the same concentration in solution of Cd, an initial pH of the solutions equal to 5, but the suspension were kept in contact for different times (15–120 min) at 25 °C. After the contact time had passed, the suspensions were filtered and the liquid was collected for analysis of the residual concentration of cadmium. Pseudo-first and second order kinetic models were applied to estimate the adsorption rate constants and the adsorbent mechanism. The influence of the initial concentration of Cd(II) ions upon the adsorption capacity of Co–Gly and Ni–Gly was measured using the same S:L ratio at different initial concentrations (range: 5–300 mg/L). The non-linearized isotherm models of Langmuir, Freundlich, and Redlich-Peterson were employed to correlate the experimental adsorption data. Their adsorption capacities have been studied as a function of pH, contact time and cadmium initial concentration. 

The studied CNs were regenerated with HCl solution 0.2 M having an initial pH = 2. For the recovery of Cd ions from the CNs surface, a S:L ratio of 1 g/L was used, and the samples were mixed for 15 min. After the regeneration process the phases were separated the recycled adsorbent was used in other adsorption process and the extracted Cd ions from the solution were determined. The materials were used in five adsorption-desorption process cycles.

### 2.4. Computational Studies

Two coordination networks—Ni–Gly and Co–Gly—containing the basic units [Ni(HO_3_PCH_2_)_2_N(H)CH_2_COO)(H_2_O)_2_] and [Co(HO_3_PCH_2_)_2_N(H)CH_2_COO)(H_2_O)_2_], respectively, were built and visualized with the aid of Mercury 4.1.3 software (Cambridge Crystallographic Data Centre, Cambridge, UK) [30]. The generated CNs were geometrically optimized by means of the semi-empirical PM3-RHF method implemented in HyperChem version 7.52 (Hypercube, Inc., Gainesville, FL, USA) [31] software. Polak-Ribiere conjugate gradient algorithm and a RMS gradient norm limit of 10^−2^ kcal/A were used, while the self-consistent field (SCF) convergence criterion was considered 10^−5^. Maestro version 12.0.012 from the Schrodinger package was used for the computation of the surface areas. [https://www.schrodinger.com/maestro].

## 3. Results and Discussion

### 3.1. Materials Characterization

**Figure 1:**
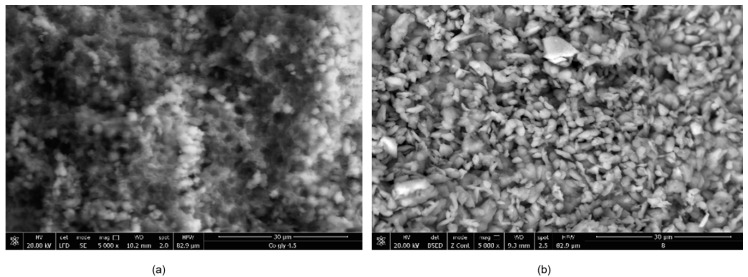
Scanning electron microscopy (SEM) images of the synthesized coordination networks (CNs) (**a**) Co–Gly; (**b**) Ni–Gly.

The morphology of the synthesized CNs is presented in Figure 1. It can be observed that the CNs based on Ni ions present a more ordered structure, with particles of well-defined sizes and shapes compared with the CN based on Co ions, which surface is more compact with particle conglomerates of various sizes and shapes. 

The specific surface area and the pore volume of the synthesized CNs are presented in Table 1. It can be observed that the Ni–Gly sample presents a higher specific surface area and a higher pore volume compared with Co–Gly sample. In accordance with the results of the characterization studies, due to its structure and morphology, it is expected that Ni–Gly to develop higher adsorption capacity in the removal process of Cd ions from aqueous solutions compared to Co–Gly sample. 

**Figure 2:**
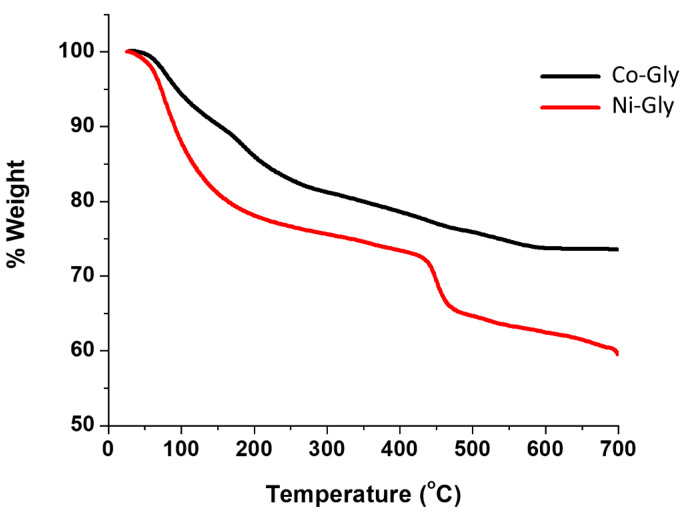
Thermal behaviour of Co–Gly and Ni–Gly.

TGA data for Ni–Gly (Ni(C_4_H_9_O_8_NP_2_)·2H_2_O) and Co–Gly (Co(C_4_H_9_O_8_NP_2_)·2H_2_O) shows that the removal of water molecules starts almost immediately upon heating and is lost slowly between 290 °C and 370 °C followed by decomposition at ~400 °C. The total weight loss caused by decomposition of all the organic composition of Ni–Gly and Co–Gly is around 35% for the former and 38% in case of Co–Gly and ocurrs at approximately 700 °C, suggesting an endothermic process (Figure 2). Detailed X-Ray Diffraction (XRD)characterisation studies for Co–Gly and Ni–Gly are under way.

### 3.2. The pH Influence upon the Adsorption Studies

**Figure 3:**
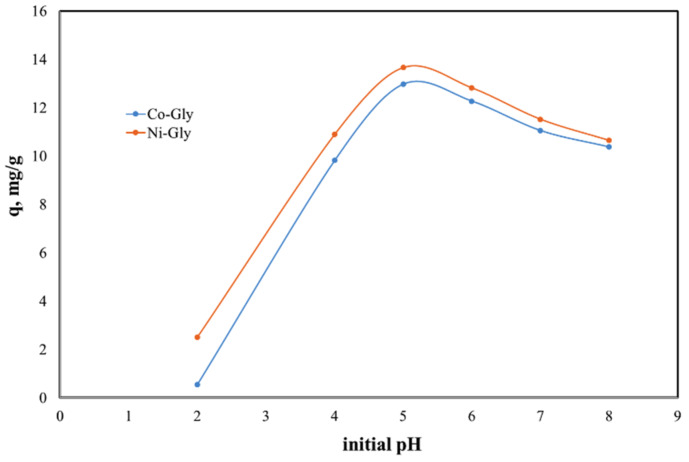
pH effect upon the adsorption capacity of the studied materials in the removal process of Cd(II) ions from aqueous solutions.

The solutions’ pH affects the properties and the degree of protonation of the adsorbent surface. Due to the fact that at higher values of pH the Cd(II) ions could precipitate the studies were carried out in the 2–8 pH range. The experimental data regarding the dependence of Cd(II) ions adsorbed by the studied materials as a function of the initial pH of the solutions are presented in Figure 3.

The initial pH of Cd(II)-containing solutions has a significant effect upon the adsorption performance of the studied materials, displaying a maximum adsorption capacity at an initial pH of 5. The adsorption capacity decreases with the change of pH around this value. This behavior can be explained by the surface loading of the adsorbent material and by the competitive adsorption of protons [17,32]. At lower pH values the adsorbent surface is positively charged and therefore there is an electrostatic repulsion between the adsorbent surface and cadmium cations [33,34]. In the same time at higher pH values, Cd ions could precipitate under Cd(OH)_2_ and then the adsorption is inhbitated [33,35,36]. Further experiments were carried out with Cd(II) solution having an initial pH of 5.

### 3.3. Kinetics of Adsorption

**Figure 4:**
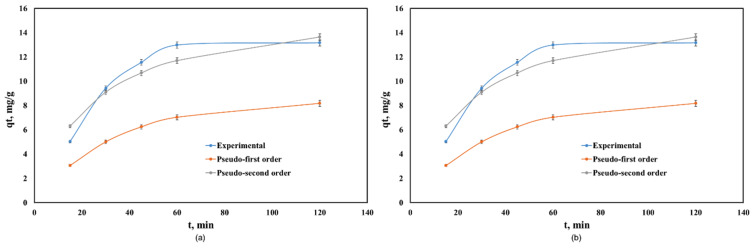
Kinetics of Cd(II) adsorption onto: (**a**) Co–Gly and (**b**) Ni-Gly.

Figure 4 shows the kinetics of Cd(II) adsorption onto Co–Gly and Ni–Gly, respectively. Adsorption is fast, in both cases, and the equilibrium between the adsorbent and adsorbate was achieved after 60 min. The kinetic data were fitted by non-linear regression using the Lagergren (pseudo-first order kinetic model) and Ho and McKay (pseudo-second order kinetic model) equations [14,17]. Table 2 presents the calculated parameters and the correlation coefficients obtained after fitting.

The regression coefficients *R*^2^ showed that the Ho and McKay model fitted the kinetic behaviour of the process when Co–Gly and Ni–Gly were used as adsorbents. For both adsorbents, the adsorption capacities calculated at equilibrium are in agreement with those experimental values obtained. The adsorption of Cd(II) ions onto Co–Gly and Ni–Gly has a chemo-sorption profile.

### 3.4. Equilibrium of Adsorption

**Figure 5:**
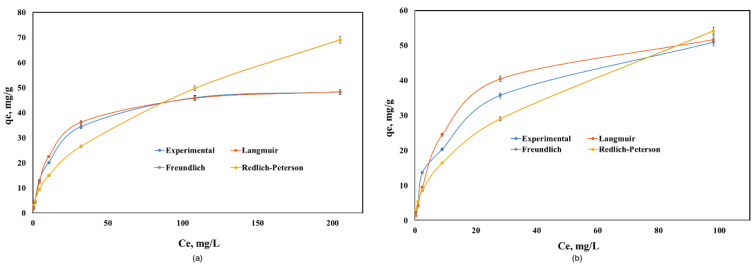
Equilibrium of Cd(II) adsorption onto: (**a**) Co–Gly and (**b**) Ni–Gly.

The equilibrium adsorption data of Cd(II) onto Co–Gly and Ni–Gly were analyzed by using the Langmuir, Freundlich and Redlich-Peterson models and non-linear analysis in order to predict the overall adsorption behavior. The isotherm parameters obtained after fitting the experimental data for the adsorption of Cd(II) on the two materials are presented in Table 3. Figure 5 presents the experimental data and the isotherms obtained by simulations of the mathematical models used.

As seen in Table 3, the Langmuir model fitted the data the best over the whole concentration range. The maximum adsorption capacities experimentally obtained were 48.2 mg/g for Cd(II) adsorption onto Co–Gly and 55 mg/g for Cd(II) adsorption onto Ni–Gly, respectively. These values are close to the maximum adsorption capacities obtained when the data are fitted by a Langmuir model (q_max_ = 51.5 mg/g for Co–Gly and 58.1 mg/g for Ni–Gly). From Table 3 it can be observed that the non-homogeneity factor n in Redlich-Peterson model has values closes to 1, so that the behavior of the samples obeys a Langmuir model. The Langmuir isotherm idea involves a monolayer coverage of adsorbate above a homogeneous adsorbent surface [17]. The essential characteristics of the Langmuir isotherm is communicated in relations of a dimensionless constant separation factor RL that is specified by the following Equation (2):

(2) $$R_{L}=\frac{1}{1+K_{L}C_{0}}$$

where *K_L_* is the Langmuir constant and *C*_0_ is the initial concentration of Cd(II) ions. The value of the separation parameter *R_L_* offers important data about the type of adsorption. The value of R_L_ point out the category of Langmuir isotherm to be irreversible (*R_L_* = 0), favorable (0< *R_L_*< 1), linear (*R_L_* = 1), or unfavorable (*R_L_* > 1) [32]. The R_L_ was established to be between 0 and 1 for the entire concentration interval, and for both studied material which indicates the favorable adsorption of cadmium onto the studied materials.

**Figure 6:**
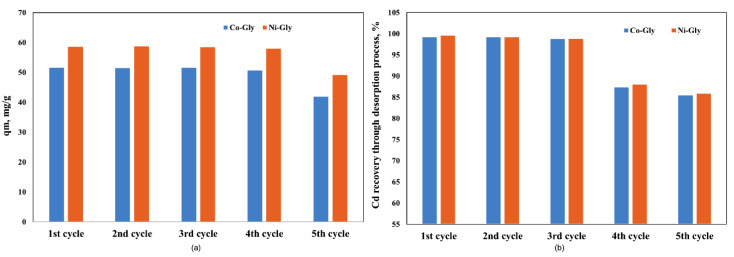
The adsorption performance of the studied CNs in various adsorption-desorption cycles (**a**) the adsorption capacity, after each cycle (**b**) Cd recovery, after each cycle.

The studied CNs were used in five adsorption-desorption process cycles and it was observed that their adsorption capacity remains constant for four adsorption-desorption cycles, then it decreases by 20% because the recovery of Cd ions from the CNs’ surface decreases (Figure 6). A decreasing Cd ion recovery capacity means that the available sites for adsorption decrease, and for this reason the adsorption capacity decreased after four adsorption-desorption process cycles. 

The maximum adsorption capacity achieved by the studied materials in the removal process of Cd(II) ions from aqueous solutions were compared with the maximum adsorption capacities obtained using other adsorbents and reported in the specialty literature. The results are presented in Table 4. It can be observed that the coordination networks present a higher efficiency in the removal process of Cd(II) ions from aqueous solutions than other low cost adsorbent materials. It could be observed that the CN-based materials reported until now in the literature, displayed higher adsorption capacities in the removal process of Cd ions from aqueous solutions, but in these cases they involved some expensive composite materials, not only CNs. Therefore, a synergistic effect of the synthesized CNs and other materials such as nanoporous carbon or iron oxide nanoparticles from the composite structures could be proposed.

### 3.5. Computational Semiempirical Studies

**Figure 7:**
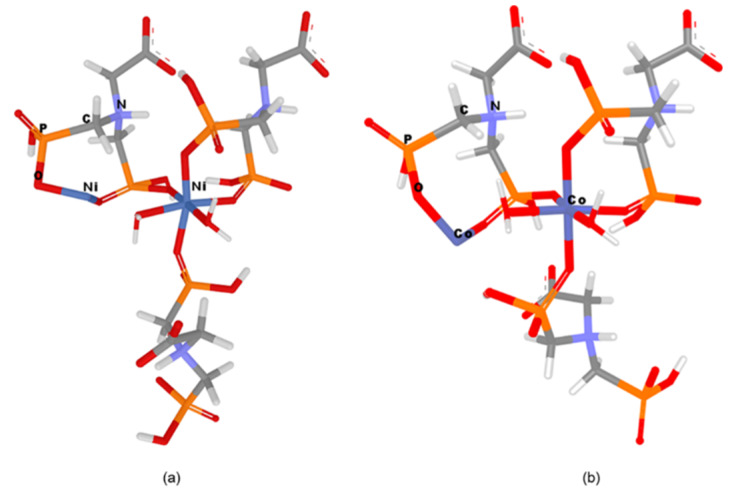
Models representation (**a**) Ni–Gly; (**b**) Co–Gly.

In order to design and geometrically optimize the following CN models: [Ni_2_((HO_3_PCH_2_)_2_N(H)CH_2_COO))_3_*2H_2_O]^2−^ and [Co_2_((HO_3_PCH_2_)_2_N(H)CH_2_COO))_3_*2H_2_O]^2−^ in silico methods were applied. The optimized structures of these networks are presented in Figure 7. 

**Figure 8:**
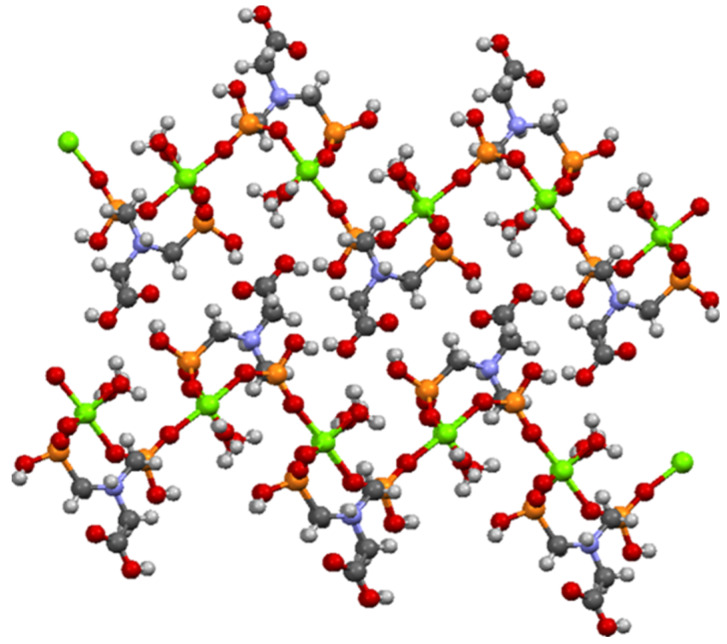
Partial view of two adjacent layers in the crystal structure of Mg–Gly model structure.

*N*,*N*′-bis-phosphonomethylglycine (Gly) in a coordination complex with Mg ion has been synthesized and structurally characterized by Demadis and co-workers [44] and was used as model structure (CCDC Reference Code 729893). *N*,*N*′-bis-phosphonomethylglycine (Gly) in coordination complex with Mg present a 2D layered architecture [44] as can be seen in Figure 8.

From Figure 8 it can be seen that the oxygen atoms belonging to the carboxylate and phosphonate groups hold the layers together by H bonding. Each layer is formed by Mg−O (phosphonate) bonds. It has been observed that the slightly distorted octahedral geometry of the Mg central ion is also maintained in the case of Ni/Co networks. The Ni^2+^/Co^2+^ ions coordinate with six oxygen atoms, two belonging to water molecules and four pertaining to phosphonate groups of the Gly ligands (Figure 7). The two axially-oriented oxygen atoms belong to phosphonate moieties, while of the oxygen atoms occupying the four equatorial positions of the octahedral geometry, two belong to phosphonate moieties, and the other two appertain to water molecules (Figure 7). As can be seen in Figure 7, the networks have rings (cavities) consisting of eight atoms; one metal ion (Co^2+^ or Ni^2+^) and one protonated nitrogen binding between them two -O-PO_2_H-CH_2_ radicals. The network cavities may confer various practical application to these materials, such as, gas storage or different atoms/molecules adsorption properties.

**Figure 9:**
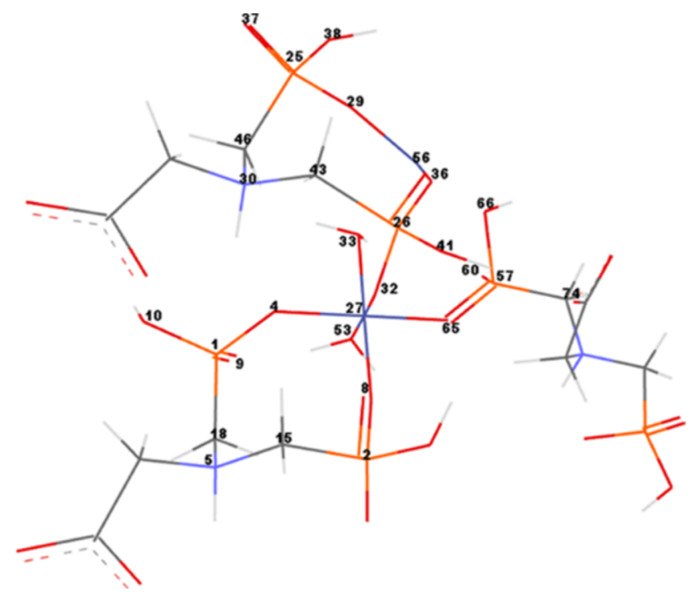
Co–Gly atoms numbering. For simplification only the numbers of significant atoms around the metal ions were represented.

In order to measure and compare the geometrical parameters of the CNs, the most important atoms of the networks were numbered (Figure 9). Co–Gly is exemplified and the atom’s numbers are equivalent for all considered networks. Bond lengths/angles, torsion angles and partial charges for the numbered atoms of the Ni–Gly and Co–Gly are presented in Table 5 and Table 6.

Considering the crystallographic structure of Mg complexed with *N*,*N*′-bis-phosphonomethyl-glycine (Mg–Gly) (Figure S1) as the most similar network to our models, some of their geometrical features were analyzed and compared. Thus, the bond length values for Me-O are in the range of 1.8–2.0 Å for Ni and Co compared with 2.0–2.1 Å resulted for Mg (Table S1). The O=P bond (from the cycle) has differences from up to 0.3 Å between Co and Mg; and up to 0.26 Å, respectively, for Ni and Mg CNs. The tendency of the length differences remains about the same for O-P bond (from the ring), shrinking the difference between Co–Gly and Mg–Gly to 0.14 Å. On the other hand, The O=P bond (from the outer phosphonate group) presents similar values in a range of 1.48–1.50 Å for all three networks. the calculated N–C and P–C bond lengths of around 1.5 and 1.9 Å, respectively, are also quite similar to the experimentally determined ones.

There is evidence of the importance of the atomic charges for modeling metal-organic frameworks [45]. The electrostatic attraction between the negatively charged CNs (Me–Gly) and the positively charged heavy metal ions (Cd^2+^) is the main factor that causes adsorbtion [46]. Due to the charge transfer from oxygen atoms to metal ions, Ni and some Co ions tend to have negative partial charges (Table 5 and Table 6). Thus, Ni27 and Ni56 ions have negative partial charge values of −0.555 and −0.594, respectively. As one can see, a negative value of −0.0996 was obtained for Co56, while the central ion Co27 has a positive charge of 0.075 (Table 5 and Table 6). 

The metal ions (Me56) which belong to the marginal rings of eight atoms are in particular the ones with negative charges and they present increased chances of giving up electrons. The surface areas for these both marginal cycles (of Ni–Gly and Co–Gly) were computed and the results show a higher value of 39.924 for Ni–Gly compared with 30.547 obtained for Co–Gly. Taking into account these informations and compare it with the absorbance affinity of Co–Gly and Ni–Gly for Cd(II) ions we can assign this effect to the accentuated negativity of the both Ni ions present in the network higher than the central Co ions as well as due to the bigger area surface for the marginal Ni-ring.

A plethora of potential properties such as adsorption, gas storage, heterogeneous catalysis, separation, ion exchange, magnetism, and sensors, can be explained by the negative charge values of the ions in these CN materials [46,47].

The adsorption efficiency of CNs is well corelated with surface area, pore size and distribution. [21]. In order to evaluate the ability of a chemical structure to donate electrons, the investigation of the highest occupied molecular orbitals (HOMO) values and their localization is of great interest.

**Figure 10:**
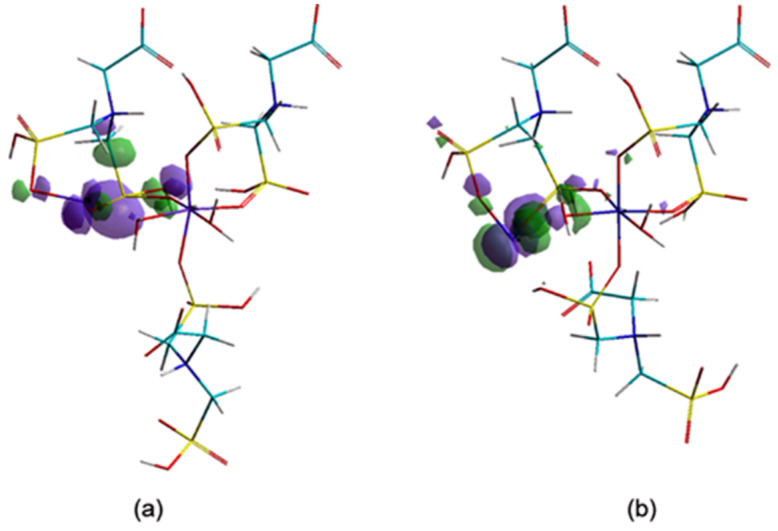
The highest occupied molecular orbital (HOMO) components (**a**) Ni–Gly; (**b**) Co–Gly.

From the orbitals component analysis (Figure 10) it can be observed that the HOMOs are located over the marginal cycles of eight atoms, mainly on metal ions (Me56). These findings are in accordance with the aforementioned results, which attested that negative charges of the Co56/Ni56 ions present increased chances of giving up electrons to the adsorbants. The Langmuir isotherm concept adopts monolayer coverage of adsorbate above a homogeneous adsorbent surface.

A series of electronic properties (heat of formation, free energy, vibrational zero-point energy, minimum and maximum fundamental vibrations, frontier orbitals and the energy gap between them) resulted from semiempirical PM3 calculations are presented in Table 7. The positive values of fundamental vibration (ν_min_) regarding both CN models show that these semiempirical calculated geometries are not transition states, confirming their stability.

The energy gap between the frontier orbitals (LUMO and HOMO) can be used to estimate the strength and stability of coordination networks. A significant band gap correlates with structural and kinetic stability [48]. Another measure for chemical stability is the heat of formation (∆*H_form_*), the lower is the value, the more stable is the complex. Analyzing Table 7 it can be observed that these calculated properties advocates for a good stability of the models, in special for Co–Gly network. Like other studies [49], our findings show that the semiempirical methods are valuable tools to be used in adsorption processes involving CNs.

## 4. Conclusions

In this study, CNs were used as adsorbent materials for the removalof Cd(II) ions from aqueous solutions. The present investigations showed that the studied CNs have a good affinity for the removal of Cd(II) ions from aqueous solution compared with other materials reported in the specialty literature. By applying suitable kinetic models to the experimental data it was established that the adsorption of Cd(II) ions on studied material is defined by a pseudo-second-order kinetic model. The equilibrium sorption data were modeled using Langmuir, Freundlich, and Redlich-Peterson isotherms and the first one provided an excellent fit of the experimental data, giving a maximum adsorption capacity of 51.5 mg/g and 58.1 mg/g for Co–Gly and Ni–Gly, respectively. 

The higher specific surface area and pore volume of Ni–Gly together with the higher negative partial charges of Ni in the network shown by PM3 semiempirical computations increase the electrostatic attraction between the Ni–Gly and the positively charged heavy metal ions (Cd^2+^). This finding is the key factor that causes higher adsorbtion capacity for Ni–Gly. Thus, Ni–Gly is the adsorbant material with a greater adsorbance capacity, as confirmed both by experimental and theoretical methods.

## Figures and Tables

**Table 1:** Specific surface area and pore volume of the synthesized CNs.

Adsorbent	Specific Surface Area, m^2^/g	Pore Volume, cm^3^/g
Co–Gly	32	0.25
Ni–Gly	45	0.85

**Table 2:** Kinetic and statistic parameters for the kinetic models.

Adsorbent	*q_exp_*	Lagergren Model $q_{t}=q_{e}(1-e^{-k_{1}t})$			Ho and McKay Model $q_{t}=q_{e}\left(1-\frac{1}{k_{2}q_{e}t+1}\right)$		
		*q_e_* (mg/g)	*k*_1_ (1/min)	*R* ^2^	*q_e_* (mg/g)	*k*_2_ (g/mg·min)	*R* ^2^
Co–Gly	13.2	8.39	0.0302	0.8146	16.36	2.5 × 10^-3^	0.9968
Ni–Gly	15.2	10.76	0.0357	0.9848	18.14	1.11 × 10^-3^	0.9968

**Table 3:** Equilibrium adsorption isotherm parameters for Cd adsorption.

Adsorbent	Langmuir Model $q_{e}=q_{max}\frac{K_{L}\cdot C_{e}}{1+K_{L}\cdot C_{e}}$				Freundlich Model $q_{e}=K_{F}\cdot C_{e}^{1/n}$			
	*q_max_* (mg/g)	*K_L_*		*R* ^2^	*K_F_*	1/*n*		*R* ^2^
Co–Gly	51.5	0.0739		0.9994	4.47	0.5143		0.9391
Ni–Gly	58.1	0.0818		0.9968	5.54	0.4972		0.9406
**Adsorbent**	**Redlich-Peterson Model** $q_{e}=q_{max}\frac{K_{RP}\cdot C_{e}}{1+{\left(K_{RP}\cdot C_{e}\right)}^{n}}$							
	***q_max_* (mg/g)**		***K_RP_***		***n***		***R*^2^**	
Co–Gly	0.485		4.47		1.02		0.9312	
Ni–Gly	0.503		5.54		0.952		0.9419	

**Table 4:** Maximum adsorption capacities developed by the various adsorbent in the removal process of Cd(II) from aqueous solutions.

Adsorbent	*q_m_*, mg/g	pH	References
Orange Peels	4.9	5	[37]
Kaolinite	7.407	8	[38]
Metakaolinite	9.174	8	[39]
Cyclodextrin metal-organic framework based nanoporous carbon	140.85	7	[24]
Sulfonated metal organic framework loaded on iron oxide nanoparticles	163.9	3	[25]
Grass char	115.8	6.8	[39]
Kaolin	1.46	6.8	[40]
Sediments	10.01	5.5	[41]
Zerovalent iron particles	714.3	-	[42]
Natural cheese	5.12	6	[43]
Co–Gly	51.5	5	Present paper
Ni–Gly	58.1	5	

**Table 5:** Geometric properties of the Ni–Gly model.

Ni–Gly								
Atom	ID	Charge	Bond	Distance	Bond Angle	Degree	Torsion Angle	Degree
Carbon	C15	−0.723	Ni56-O29	1.815	O29-Ni56-O36	94.636	C46-P25-O29-Ni56	9.656
Carbon	C18	−0.697	Ni56-O36	1.792	Ni56-O36-P26	138.283	P25-O29-Ni56-O36	−92.872
Carbon	C43	−0.599	O36-P26	1.853	O36-P26-C43	93.346	O29-Ni56-O36-P26	130.169
Carbon	C46	−0.807	P26-C43	1.986	P26-C43-N30	124.21	Ni56-O36-P26-O32	41.057
Carbon	C74	−0.763	C43-N30	1.493	C43-N30-C46	116.868	O36-P26-O32-Ni27	1.422
Hydrogen	H34	0.2853	N30-C46	1.502	N30-C46-P25	130.108	P26-O32-Ni27-O4	−94.052
Hydrogen	H35	0.2188	C46-P25	1.935	C46-P25-O29	113.781	P26-O32-Ni27-O8	170.186
Hydrogen	H54	0.2133	P25-O37	1.467	P25-O29-Ni56	95.464	P26-O32-Ni27-O33	−2.211
Hydrogen	H55	0.2568	P25-O38	1.700	O4-Ni27-O8	97.744	P26-O32-Ni27-O65	69.181
Nitrogen	N5	0.6925	P26-O41	1.766	O8-Ni27-O53	89.307	O32-Ni27-O4-P1	−150.244
Nitrogen	N30	0.7692	P26-O32	1.786	O53-Ni27-O65	85.753	O32-Ni27-O8-P2	28.512
Nickel	Ni27	−0.555	O32-Ni27	1.842	O65-Ni27-O33	71.3	Ni27-O8-P2-C15	53.962
Nickel	Ni56	−0.594	Ni27-O4	1.869	O33-Ni27-O32	93.215	O4-Ni27-O8-P2	−62.239
Oxygen	O4	−0.567	O4-P1	1.705	O32-Ni27-O4	90.604	O32-Ni27-O65-P57	70.339
Oxygen	O7	−0.835	P1-O9	1.492	O4-Ni27-O53	91.125		
Oxygen	O8	−0.433	P1-O10	1.634	O4-Ni27-O65	162.982		
Oxygen	O9	−0.886	P1-C18	1.912	O4-Ni27-O33	91.804		
Oxygen	O10	−0.670	C18-N5	1.499	O8-Ni27-O65	100.934		
Oxygen	O13	−0.700	N5-C15	1.494	O8-Ni27-O33	171.094		
Oxygen	O29	−0.476	C15-P2	1.898	O8-Ni27-O32	91.428		
Oxygen	O32	−0.323	P2-O7	1.456	O53-Ni27-O33	85.817		
Oxygen	O33	0.2309	P2-O13	1.634	O53-Ni27-O32	178.044		
Oxygen	O36	−0.415	P2-O8	1.743	O65-Ni27-O32	92.328		
Oxygen	O37	−0.873	Ni27-O8	1.836				
Oxygen	O38	−0.699	Ni27-O53	1.925				
Oxygen	O41	−0.650	O53-H54	0.959				
Oxygen	O53	−0.085	O53-H55	0.986				
Oxygen	O60	−0.852	Ni27-O33	1.947				
Oxygen	O65	−0.604	O33-H34	1.012				
Oxygen	O66	−0.692	O33-H35	0.979				
Phosphorus	P1	2.0639	Ni27-O65	1.853				
Phosphorus	P2	2.0923	O65-P57	1.733				
Phosphorus	P25	2.1319	P57-O60	1.450				
Phosphorus	P26	1.1473	P57-O66	1.683				
Phosphorus	P57	2.0932	P57-C74	1.848				

**Table 6:** Geometric properties of the Co–Gly model.

Co–Gly								
Atom	ID	Charge	Bond	Distance	Bond Angle	Degree	Torsion Angle	Degree
Carbon	C15	−0.7165	Co56-O29	1.874	O29-Co56-O36	103.345	C46-P25-O29-Co56	−57.484
Carbon	C18	−0.6848	Co56-O36	2.025	Co56-O36-P26	136.025	P25-O29-Co56-O36	−10.336
Carbon	C43	−0.731	O36-P26	1.560	O36-P26-C43	104.476	O29-Co56-O36-P26	78.858
Carbon	C46	−0.7151	P26-C43	1.892	P26-C43-N30	121.608	Co56-O36-P26-O32	36.937
Carbon	C74	−0.7589	C43-N30	1.494	C43-N30-C46	114.412	O36-P26-O32-Co27	−16.624
Hydrogen	H34	0.209	N30-C46	1.503	N30-C46-P25	127.21	P26-O32-Co27-O4	−104.721
Hydrogen	H35	0.2187	C46-P25	1.956	C46-P25-O29	102.822	P26-O32-Co27-O8	159.331
Hydrogen	H54	0.2528	P25-O37	1.463	P25-O29-Co56	139.07	P26-O32-Co27-O33	−18.618
Hydrogen	H55	0.2879	P25-O38	1.707	O4-Co27-O8	96.057	P26-O32-Co27-O65	75.019
Nitrogen	N5	0.7035	P26-O41	1.641	O8-Co27-O53	83.833	O32-Co27-O4-P1	−138.862
Nitrogen	N30	0.7478	P26-O32	1.659	O53-Co27-O65	88.608	O32-Co27-O8-P2	17.287
Cobalt	Co27	0.075	O32-Co27	1.918	O65-Co27-O33	93.076	Co27-O8-P2-C15	46.317
Cobalt	Co56	−0.0996	Co27-O4	1.917	O33-Co27-O32	97.175	O4-Co27-O8-P2	−69.782
Oxygen	O4	−0.7808	O4-P1	1.675	O32-Co27-O4	86.851	O32-Co27-O65-P57	−105.659
Oxygen	O7	−0.8435	P1-O9	1.504	O4-Co27-O53	90.374		
Oxygen	O8	−0.7288	P1-O10	1.642	O4-Co27-O65	178.93		
Oxygen	O9	−0.8995	P1-C18	1.913	O4-Co27-O33	86.533		
Oxygen	O10	−0.6691	C18-N5	1.501	O8-Co27-O65	84.167		
Oxygen	O13	−0.6707	N5-C15	1.493	O8-Co27-O33	170.364		
Oxygen	O29	−0.7278	C15-P2	1.921	O8-Co27-O32	92.241		
Oxygen	O32	−0.7066	P2-O7	1.461	O53-Co27-O33	86.876		
Oxygen	O33	−0.0615	P2-O13	1.68	O53-Co27-O32	174.936		
Oxygen	O36	−0.7897	P2-O8	1.669	O65-Co27-O32	94.188		
Oxygen	O37	−0.8422	Co27-O8	1.888				
Oxygen	O38	−0.6909	Co27-O53	1.972				
Oxygen	O41	−0.6614	O53-H54	0.965				
Oxygen	O53	−0.2364	O53-H55	0.995				
Oxygen	O60	−0.8173	Co27-O33	2.000				
Oxygen	O65	−0.7366	O33-H34	0.978				
Oxygen	O66	−0.5273	O33-H35	0.973				
Phosphorus	P1	2.0692	Co27-O65	1.936				
Phosphorus	P2	2.0995	O65-P57	1.632				
Phosphorus	P25	2.0392	P57-O60	1.451				
Phosphorus	P26	1.9959	P57-O66	1.823				
Phosphorus	P57	2.0514	P57-C74	1.853				

**Table 7:** The electronic properties of the CNs models.

CNs/Electronic Properties	∆*H_form_* (kcal/mol)	Free Energy (kcal/mol)	Zero-pt Vib Energy (kcal/mol)	*ν_min_* (cm^−1^)	*ν_max_* (cm^−1^)	HOMO (eV)	LUMO (eV)	LUMO—HOMO (eV)
Co–Gly	−1990.3819	−284904	342.5893	16.06	3959.68	−4.7667	3.2892	8.0559
Ni–Gly	−1828.6042	−296474	345.3689	9.67	3963.54	−4.6634	2.7312	7.3946

## Supplementary Materials

The following are available online at https://www.mdpi.com/2079-4991/10/5/899/s1, Figure S1: Superposition of the crystal structure Mg-Gly over the optimized structure of Mg-Gly, Table S1: Geometric properties of the Mg-Gly model.
