# Supplementary material for: New Efficient Adsorbent Materials for the Removal of Cd(II) from Aqueous Solutions
Source: Nanomaterials (Basel). 2020 May 8;10(5):899. doi: 10.3390/nano10050899 (PMC7279495; doi:10.3390/nano10050899)
Supplement: Supplementary file 1 [file nanomaterials-10-00899-s001.pdf]

nanomaterials

Supplementary Information

New Efficient Adsorbent Materials for the Removal of Cd(II) from Aqueous Solutions

Aurelia Visa [1], Bianca Maranescu [1], Lavinia Lupa [2], Luminita Crisan [1] and Ana Borota [1]*

[1] "Coriolan Dragulescu" Institute of Chemistry, 24 M. Viteazul Ave, Timişoara - 300223, Romania,

[2] Faculty of Industrial Chemistry and Enviromental Engineering, University Politehnica Timisoara, 2 Piata Victoriei, 300006, Timisoara, Romania

* Correspondence: ana_borota@acad-icht.tm.edu.ro

Received: date; Accepted: date; Published: date

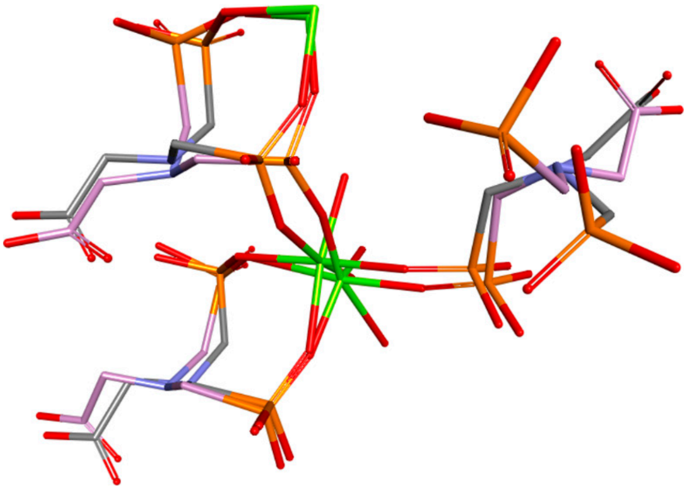

Figure S1. Superposition of the crystal structure of Mg-Gly (carbon atoms in grey) over the optimized structure of Mg-Gly (carbon atoms in purple) with PM3 semiempirical method. The RMSD between them is of 1.219 for heavy atoms.

nanomaterials

MDPI

Table S1. Geometric properties of the Mg-Gly model (CS - Crystal structure; OS - Optimized structure)

Bond	Distance			Bond Angle	Degree			Torsion Angle	Degree		
	CS	OS	Residuals		CS	OS	Residuals		CS	OS	Residuals
57-O66	1.561	1.715	-0.154	O29-Mg56-O36	89.442	109.344	-19.902	C46-P25-O29-Mg56	66.587	-24.389	90.976
P57-O60	1.505	1.471	0.035	Mg56-O36-P26	151.054	156.611	-5.557	P25-O29-Mg56-O36	-77.663	-2.868	-74.795
P57-C74	1.822	1.958	-0.136	O36-P26-C43	110.219	105.944	4.275	O29-Mg56-O36-P26	-30.074	-10.206	-19.868
P2-O8	1.485	1.590	-0.105	P26-C43-N30	115.200	124.237	-9.037	Mg56-O36-P26-O32	147.757	118.695	29.062
P2-O7	1.496	1.474	0.022	C43-N30-C46	115.746	114.588	1.158	O36-P26-O32-Mg27	-36.196	-123.688	87.492
P2-O13	1.577	1.693	-0.116	N30-C46-P25	120.139	129.166	-9.027	P26-O32-Mg27-O4	-82.983	24.247	-107.230
P26-O41	1.577	1.637	-0.061	C46-P25-O29	108.631	106.910	1.721	P26-O32-Mg27-O8	-171.513	-56.855	-114.658
P26-O32	1.496	1.521	-0.025	P25-O29-Mg56	132.492	150.599	-18.107	P26-O32-Mg27-O33	12.571	100.428	-87.857
P26-C43	1.831	1.877	-0.046	O4-Mg27-O8	89.442	85.255	4.187	P26-O32-Mg27-O65	99.401	-167.967	267.368
P25-O38	1.561	1.694	-0.133	O8-Mg27-O53	89.928	70.351	19.577	O32-Mg27-O4-P1	-178.943	172.126	-351.069
P25-O37	1.480	1.466	0.014	O53-Mg27-O65	92.418	70.148	22.270	O32-Mg27-O8-P2	55.192	35.097	20.095
P1-O9	1.480	1.484	-0.004	O65-Mg27-O33	86.855	88.898	-2.043	Mg27-O8-P2-C15	23.384	33.866	-10.482

P1-O10	1.561	1.724	-0.163	O33-Mg27-O32	89.632	109.699	-20.067	O4-Mg27-O8-P2	-30.075	-45.615	15.540
P1-C18	1.822	1.959	-0.137	O32-Mg27-O4	85.465	84.555	0.910	O32-Mg27-O65-P57	-119.670	16.094	-135.764
O65-P57	1.505	1.471	0.035	O4-Mg27-O53	89.329	105.720	-16.391	Mg27-O4-P1-C18	66.587	64.821	1.766
O53-H55	0.930	0.971	-0.041	O4-Mg27-O65	177.264	167.653	9.611				
O53-H54	0.929	0.970	-0.041	O4-Mg27-O33	95.508	78.936	16.572				
O4-P1	1.505	1.556	-0.051	O8-Mg27-O65	88.486	103.645	-15.159				
O36-P26	1.485	1.642	-0.157	O8-Mg27-O33	168.434	124.678	43.756				
O33-H35	0.900	0.959	-0.059	O8-Mg27-O32	101.199	121.142	-19.943				
O33-H34	0.821	0.973	-0.152	O53-Mg27-O33	80.704	63.954	16.750				
O32-Mg27	2.033	1.867	0.167	O53-Mg27-O32	168.544	165.792	2.752				
N5-C15	1.504	1.493	0.011	O65-Mg27-O32	93.183	97.732	-4.549				
N30-C46	1.511	1.521	-0.010								
Mg56-O36	2.023	1.789	0.234								
Mg56-O29	2.088	1.804	0.285								
Mg27-O8	2.023	1.861	0.163								
Mg27-O65	2.018	1.876	0.143								
Mg27-O53	2.145	2.479	-0.334								
Mg27-O4	2.088	2.500	-0.412								
Mg27-O33	2.113	1.891	0.222								

C46-P25	1.822	1.918	-0.097
C43-N30	1.504	1.499	0.005
C18-N5	1.511	1.500	0.012
C15-P2	1.831	1.952	-0.121
